# The medial ligaments and the ACL restrain anteromedial laxity of the knee

**DOI:** 10.1007/s00167-020-06084-4

**Published:** 2020-06-05

**Authors:** S. Ball, J. M. Stephen, H. El-Daou, A. Williams, Andrew A. Amis

**Affiliations:** 1 Fortius Clinic, London, UK; 2 Orthopaedic Surgery Department, Chelsea and Westminster Hospital, London, UK; 3 Biomechanics Group, Mechanical Engineering Department, Imperial College London, London, SW7 2AZ UK; 4 Musculoskeletal Surgery Group, Imperial College London School of Medicine, London, UK

**Keywords:** Anteromedial rotatory instability, Anterior cruciate ligament, Medial collateral ligament, Posterior oblique ligament, Biomechanics, Restraint of tibiofemoral joint laxity

## Abstract

**Purpose:**

The purpose of this study was to determine the contribution of each of the ACL and medial ligament structures in resisting anteromedial rotatory instability (AMRI) loads applied in vitro.

**Methods:**

Twelve knees were tested using a robotic system. It imposed loads simulating clinical laxity tests at 0° to 90° flexion: ±90 N anterior–posterior force, ±8 Nm varus–valgus moment, and ±5 Nm internal–external rotation, and the tibial displacements were measured in the intact knee. The ACL and individual medial structures—retinaculum, superficial and deep medial collateral ligament (sMCL and dMCL), and posteromedial capsule with oblique ligament (POL + PMC)—were sectioned sequentially. The tibial displacements were reapplied after each cut and the reduced loads required allowed the contribution of each structure to be calculated.

**Results:**

For anterior translation, the ACL was the primary restraint, resisting 63–77% of the drawer force across 0° to 90°, the sMCL contributing 4–7%. For posterior translation, the POL + PMC contributed 10% of the restraint in extension; other structures were not significant. For valgus load, the sMCL was the primary restraint (40–54%) across 0° to 90°, the dMCL 12%, and POL + PMC 16% in extension. For external rotation, the dMCL resisted 23–13% across 0° to 90°, the sMCL 13–22%, and the ACL 6–9%.

**Conclusion:**

The dMCL is the largest medial restraint to tibial external rotation in extension. Therefore, following a combined ACL + MCL injury, AMRI may persist if there is inadequate healing of both the sMCL and dMCL, and MCL deficiency increases the risk of ACL graft failure.

## Introduction

Combined anterior cruciate ligament (ACL) and medial collateral ligament (MCL) injury is the most common two-ligament knee injury, and is associated with anteromedial rotatory instability (AMRI) [37]. Many knee injuries that rupture the ACL include a tibial rotation [16, 32]. This mechanism is often shown by the bone bruises, which indicate the tibiofemoral joint posture during the injury that occurred [22]. Differing ACL injury mechanisms may occur, and some knees also suffer MCL incompetence, following a valgus and external rotation mechanism of injury [16]. These injuries may lead to abnormally increased tibial rotational laxity [12].

In the knee with lateral-side injury with anterolateral rotatory instability (ALRI), isolated ACL reconstruction may not return the internal rotational laxity to normal, because lateral structures are important restraints of tibial internal rotation [18]. Combined procedures such as ACL plus lateral extra-articular tenodesis can restore the internal rotation laxity to normal [15], and reduce ACL graft failure rate [9, 30].

In contrast to ALRI, there has been little recent work on the biomechanics of AMRI, which may result from valgus plus external rotation laxity [17, 29]. Although MCL injuries often heal with conservative treatment [7, 14], some do not, leading to chronic pathological laxity and knee instability that requires surgery [20, 36–38]. If the injury is unaddressed, it leads to an increased rate of ACL graft failure [31], and similar findings have been made with lateral-side injuries [9, 30].

**Fig. 1:**
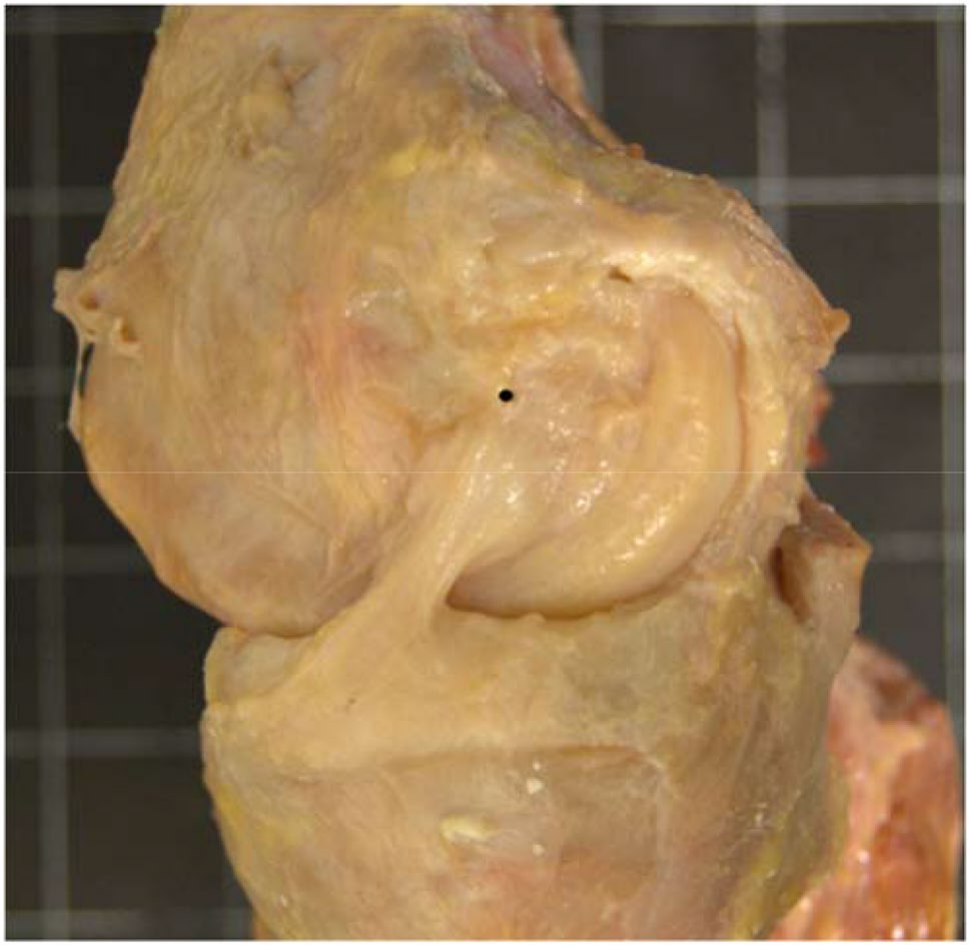
Superficial MCL (sMCL) and posteromedial capsule, which includes the posterior oblique ligament (POL), have been removed to reveal the deep MCL (dMCL). The dMCL is taut and well-aligned to resist tibial external rotation in the extended knee with tibial external rotation. The black dot is the centre of the femoral attachment of the sMCL (medial aspect of a right knee, with the anterior to the left and proximal to the top)

The medial soft-tissue complex consists principally of the superficial medial collateral ligament (sMCL), the deep medial collateral ligament (dMCL), and the posteromedial capsule (PMC) [25, 33], and they make distinct contributions to restraint of tibiofemoral joint laxity [10, 12, 24]. The PMC includes fibres that cross the joint posteromedially, and the posterior oblique ligament (POL) may be identified among them [13]. It has long been accepted that the sMCL is the primary restraint to valgus laxity [11], but those data were obtained using obsolete methods that restrained secondary coupled movements of the tibia. It might be anticipated from its orientation that the dMCL could contribute to resisting tibial external rotation (Fig. 1), and cutting the whole MCL increases tibial external rotation, whether the ACL is intact or not [12]. However, the behaviour of the knee with combined ACL/MCL injuries has been little studied, and data on the individual contributions of the soft-tissue structures to resisting AMRI remain incomplete. This raises the question of how well an isolated ACL reconstruction can restore AMRI to normal, in the presence of the combined injury, without sustaining excess loads putting it at risk of failure [31, 38]. The basic lesion present in all patients with AMRI is rupture of the dMCL, sometimes in isolation but more commonly with lesions of the sMCL and ACL [29].

Noting the previous work, it was hypothesised that a sequential cutting study of the ACL, MCL, and POL + PMC would demonstrate that the ACL provides significant restraint of tibial anterior translation, the sMCL restrains valgus angulation, and the dMCL external rotation, across the arc of knee flexion. The objective of this work was to use a modern robotic testing method to obtain tibiofemoral restraint data that would quantify the roles of the medial soft tissues in knee stability. The clinical usefulness of this is that it would show which are the structures that are most important to address in the presence of pathological laxity across the arc of knee flexion.

## Materials and methods

This study used 17 unpaired fresh-frozen cadaveric knees, 5 for a pilot study, and then 12 that are reported here: 6 male and 6 female, aged 38 ± 7 (22–48) [mean ± SD (range min–max)] years. These were obtained from a tissue bank following research ethics committee permit. After thawing 24 h at 4 °C, the knees were examined manually to check that there were no ligament laxities, no stiffness that prevented full extension, and no scars to indicate previous surgery. Examination post-testing confirmed the absence of articular cartilage erosions, although surface roughening was accepted.

**Fig. 2:**
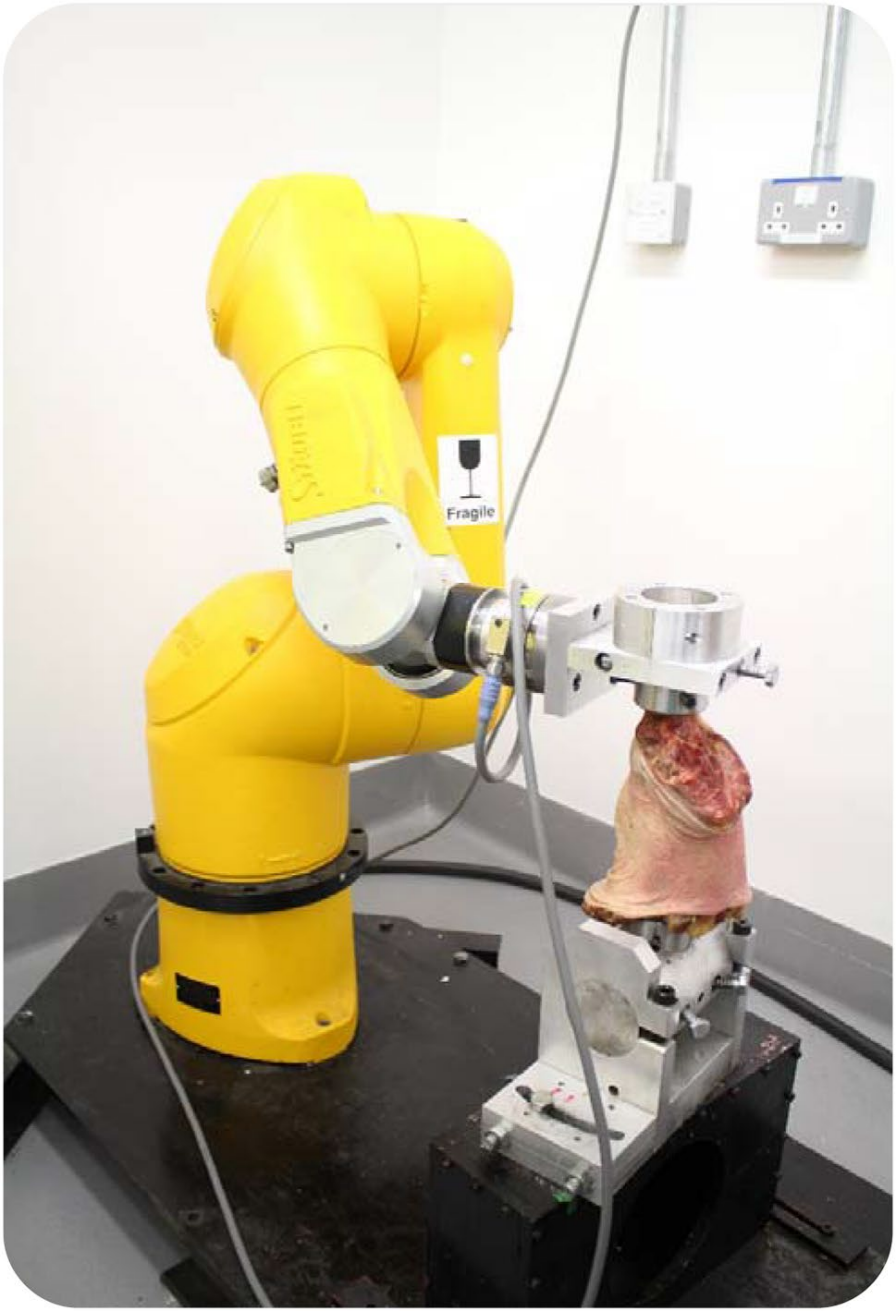
Robotic joint testing system with knee specimen in place at 0° flexion. The tibia is secured to the end of the robot arm, which has the load cell at its wrist (where the cable attaches). The femur is secured vertically in the fixed mounting on the base of the robot system

Each knee was prepared by cutting the femur and tibia to lengths of 160 and 170 mm, respectively, for uniformity of mounting in a robotic testing system. The bone ends had all soft tissue removed for a length of 60 mm and were then fixed coaxially into tubular stainless steel bone pots using poly-methylmethacrylate bone cement. The femur was aligned with the robotic testing system by cementing it with the knee fully extended and the posterior condylar axis parallel with the femoral mounting fixture. The testing system used a Staubli TX90 industrial robot (Staubli, Switzerland) with a 6 degrees-of-freedom (DoF) forces plus moments load cell (ATI Omega85, ATI Industrial Automation, Apex, USA) mounted onto the end of the moving arm of the robot. The robot had been found to have a repositioning accuracy of 0.11 mm and 0.12° [6] and the load cell had a resolution better than 0.11 N and 0.004 Nm. The femoral bone pot was secured in a stationary mounting on the baseplate of the robot system, and the tibial pot was secured to the moving load cell/robot arm (Fig. 2).

### Intact knee kinematics

Before being secured into the robot mountings, the knee was manually flexed/extended 20 times to minimise soft-tissue hysteresis. The robot then moved the tibia incrementally in the arc of flexion; after each 1° increment, the robot found the ‘neutral’ position where the forces and moments in the remaining 5 DoF were minimised, before moving further into flexion. Each ‘neutral’ position was recorded, and together, they could be played back to create the path of passive knee flexion. The robot then imposed loads to simulate clinical laxity tests at full extension, 30°, 60°, and 90° flexion. The following loads were applied to the tibia: ± 90 N anterior–posterior (AP) draw force, ± 8 Nm varus–valgus (VV) moment, and ± 5 Nm internal–external (IE) rotation torque. The robot applied the force/torque in the primary DoF, maintained the chosen flexion/extension angle, and minimised the loads in the four remaining DoF as the tibia moved. The loads were comparable to other studies of knee laxity [12, 15] with the AP force equivalent to that applied by a KT 1000™ arthrometer (MEDmetric, CA, USA) [4]. Each test was repeated three times, and the mean displacement was calculated.

### Contributions of medial soft-tissue structures

The soft tissues were cut sequentially to discern their contribution to joint stability. The ACL was cut via portals at both sides of the patellar tendon, with care taken to ensure that all fibres of the ACL were sectioned; this was confirmed with arthroscopic inspection. The medial structures were cut transversely at the level of the superior rim of the medial meniscus, while the knee remained mounted in the robot.

After each cut, the laxity tests were repeated, with the robot moving the tibia along the same path as that for the intact knee: any reduction in the force/torque required to reach the end-point position of the intact knee tests was taken to represent the contribution to tibial restraint that the cut structure had made when it was intact [26].

Tests of AP, IE, and VV laxity were performed in the following sequential states of the knee:

Intact; ACL cut; ACL + anteromedial retinaculum (AMR) cut (i.e., layers 1 and 2 immediately anterior to the anterior edge of the sMCL, but not the deeper dMCL [25, 33]); ACL + AMR + sMCL cut; ACL + AMR + sMCL + dMCL cut; ACL + AMR + sMCL + dMCL + PMC + POL cut.

The dissection of the AMR was found to be necessary in a pilot study of five knees, to allow clear identification and separation of the dMCL; data from these knees were incomplete and are unreported because of ligament damage during those dissections.

### Statistical analysis

A power calculation (G×Power v3.1.9.2, University of Kiel, Germany), based on a tibial external rotation of 22.5 ± 3.7° in a prior study [24], determined that a minimum sample size of 7 would be needed to detect a significant change in laxity of 4.0° between the intact and cut state with 80% power and 95% confidence. The results below are for 12 knees.

The following statistical analysis was performed (SPSS v22, IBM, Armonk, NY):

A Shapiro–Wilk normality test was applied to each set of data, and found that the data sets were not distributed normally, so non-parametric testing was used.

A related-samples (repeated-measures) Friedman’s two-way analysis of variance by ranks was performed to compare loads between the intact and cut states.

A Wilcoxon signed-rank test was performed to examine the force/torque contributions of each of the cut structures at each flexion angle; the significance level was set at *p* < 0.05.

A post hoc power analysis (G×Power v3.1.9.7, University of Kiel, Germany), based on restraint data obtained, found that for a change of restraint of 5 ± 3% SD, a one-way repeated-measures analysis with *p* = 0.05 gave a power >99% with 12 specimens.

## Results

### Tibial anterior–posterior translation

**Fig. 3:**
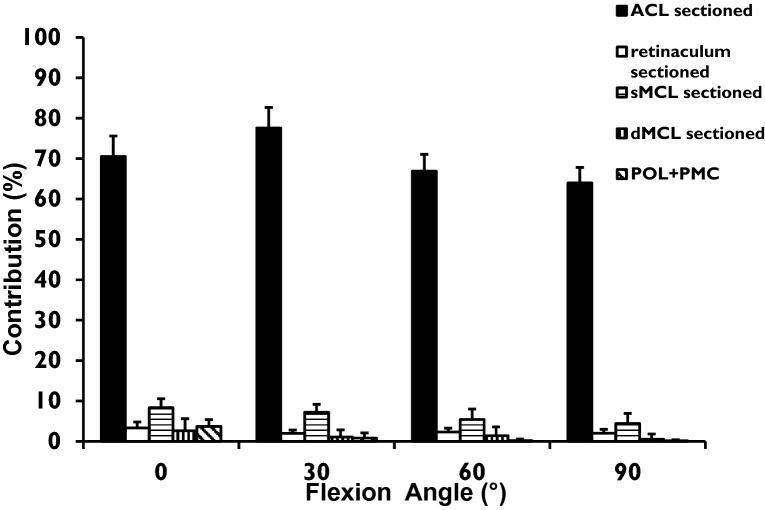
Contributions (%) of each of the ACL, AMR, sMCL, dMCL, and POL + PMC to resisting 90 N tibial anterior translation force, across 0° to 90^o^ knee flexion (mean + SD, *n* = 12). Both the ACL and sMCL were significant restraints at all angles tested

The force required to reach the anterior translation limit of the intact knee reduced significantly (*p* < 0.0001) after the sequential cutting. The ACL was the greatest single restraint to tibial anterior translation across 0° to 90° knee flexion (Fig. 3), contributing 63–78% of the restraint (*p* = 0.001 at all angles). The sMCL also contributed significantly to resisting anterior translation (*p* = 0.001 at all angles), ranging from 7 to 4%. The other structures each resisted <4% of the restraint at any angle of flexion.

A 90 N tibial posterior translation force was not resisted significantly by the ACL, sMCL, nor the dMCL. The PMC + POL, which was tight near knee extension, provided 12% of the resistance at 0° knee flexion, and 4% at 30° flexion, beyond which its contribution was negligible.

### Tibial internal–external rotation

**Fig. 4:**
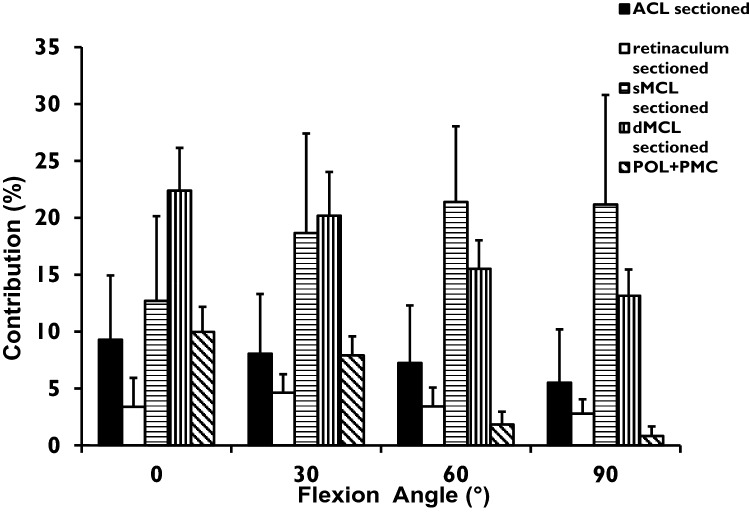
Contributions (%) of each of the ACL, AMR, sMCL, dMCL, and POL + PMC to resisting 5 Nm tibial external rotation torque, across 0° to 90^o^ knee flexion (mean + SD, *n* = 12)

The torque required to reach the external rotation limit of the intact knee reduced significantly (*p* < 0.0001) after the sequential cutting. In response to 5 Nm tibial external rotation torque, the ACL resisted 9–6% across 0° to 90° knee flexion, the sMCL resisted 13–21%, and the dMCL resisted 23–13% across 0° to 90° (all: *p* = 0.001; Fig. 4). There was a reciprocal relationship between the dMCL and sMCL as the knee flexed: the dMCL was the largest single restraint of tibial external rotation in the extended knee and the sMCL at 90° flexion. The POL + PMC also resisted tibial external rotation, ranging from 10% at 0° flexion to close to zero at 60° and 90° knee flexion.

The torque required to reach the internal rotation limit of the intact knee reduced significantly (*p* < 0.0001) after the sequential cutting. In response to 5 Nm tibial internal rotation torque, the ACL resisted 18–12% across 0° to 90° knee flexion. The POL + PMC resisted ~17% at 0° and 30° flexion, and close to 0 at 60° and 90° flexion. The sMCL resisted 13% of the internal rotation torque at 0° and 30° knee flexion, falling to 5% at 90° flexion. These contributions above 10% were significant.

### Valgus–varus angulation laxity

**Fig. 5:**
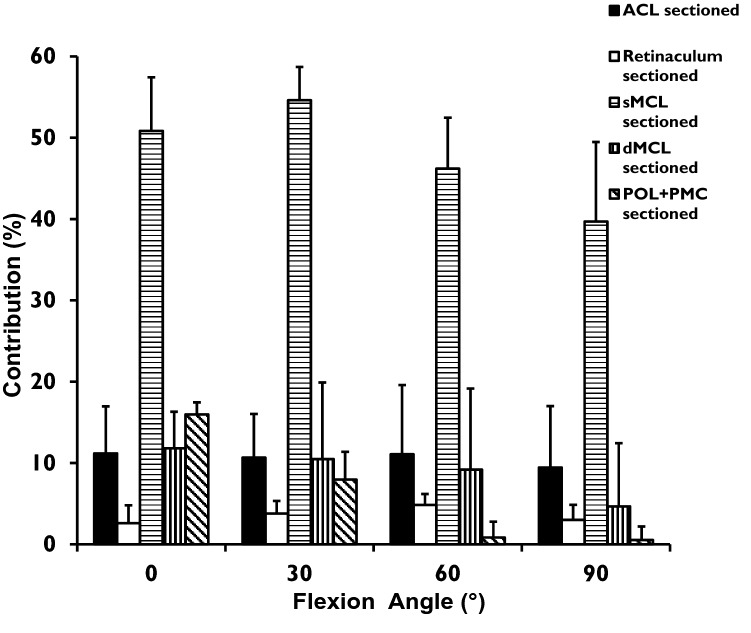
Contributions (%) of each of the ACL, AMR, sMCL, dMCL, and POL + PMC to resisting 8 Nm tibial valgus angulation moment, across 0° to 90^o^ knee flexion (mean + SD, *n* = 12)

The valgus loading required to reach the laxity limit of the intact knee reduced significantly (*p* < 0.0001) after the sequential cutting. The ACL resisted ~10% of the moment across 0° to 90° knee flexion (Fig. 5). The sMCL was the largest contributor to resisting valgus, ranging from 51 to 55% at 0° and 30° flexion, falling to 40% at 90° flexion. The contribution of the dMCL reduced from 12 to 5% across 0° to 90° knee flexion (all: *p* = 0.001). The contribution of the POL/PMC reduced from 16% at 0° (*p* = 0.001) to almost 0 at 60° and 90° knee flexion (n.s.).

Under a varus moment, none of the medial structures studied in this experiment made a measurable resistance to resisting laxity. The ACL provided ~4% of the resistance to varus.

## Discussion

The main finding of this study was that, with loads simulating those used in clinical examination, the primary medial soft-tissue ligamentous restraint to tibial external rotation was the deep MCL in the extended knee (23%). The sMCL was dominant in deeper (60° and 90°) knee flexion. The ACL and the posteromedial capsule (which included the POL) each contributed ~10% of the resistance to tibial external rotation in the extended knee, which reduced with knee flexion. These novel findings relating to AMRI were supported by data confirming the dominance of the ACL in restraining tibial anterior translation, and the sMCL restraining tibial valgus angulation, across 0° to 90° knee flexion. These findings are broadly in line with the initial hypotheses.

It has long been known that the ACL and sMCL are the primary restraints to tibial anterior translation [2] and valgus angulation [11], respectively, but those earlier works used methods that inhibited secondary DoF of tibial motion, such as internal–external coupled rotation. That limitation would have affected the relative contributions of the medial soft tissues, and it is overcome by use of a robotic system that can minimise secondary loads in real time when it displaces the tibia away from its resting neutral position. Hence, we might expect the data from the present study to be more representative of clinical examination. Since those classic papers, there has been little objective evidence of the contributions of the medial soft-tissue structures to restraint of tibial external rotation and AMRI. Many papers have reported changes of knee laxity after cutting ligaments [10, 34], but those laxity changes do not provide data on the contribution to restraint provided by the ligaments, to stabilise the knee. That is the data provided in the present paper, which identifies the primary restraints.

Slocum and Larson [29] reported that rupture of the dMCL was the basic lesion, which permits abnormal tibial external rotation, and that the injury mechanism included tibial external rotation and valgus when the knee was flexed 90°. They also noted that the medial injury led to the axis of tibial axial rotation moving into the lateral compartment, which leads to the characteristic prominence of the anteromedial aspect of the tibial plateau during an anterior draw test of the externally rotated knee. This has become known as the ‘Slocum and Larson test’ for AMRI. Slocum and Larson noted that increasing laxity resulted from progressive failure of the sMCL and then the ACL, and that AMRI could be present without the ACL being ruptured. Kennedy and Fowler [17] found that 30° of tibial external rotation did not damage the knee, that the dMCL failed at ~45° external rotation, and further rotation ruptured the sMCL and then the ACL.

The importance of the dMCL in the control of tibial external rotation might be unexpected, in view of its relatively small size and its failure strength being less than that of the sMCL [23]. However, we have found that the fibres of the dMCL are oriented antero-distally from the femur when the tibia is externally rotated, and are obviously stretched by that motion [35] (Fig. 1). The dMCL is a capsular structure oriented antero-distally across the joint line and overlaid by the stronger axially aligned sMCL. This is reminiscent of the antero-distally oriented anterolateral ligament (ALL) overlaid by the ilio-tibial band at the lateral side of the knee [3, 5, 8, 19]. Therefore, the dMCL may be an equivalent to the ALL at the medial aspect-arguably an “AML”?

Clinical observations of chronic dMCL injury [21] have included pain and disability among football (soccer) players when their knees are loaded rapidly in external rotation, for example when kicking the ball with the medial side of the forefoot. This suggests that development of dMCL reconstructions, either in isolation or in combination with sMCL and ACL reconstruction, will be beneficial for patients in this scenario but also for AMRI. For conservative treatment to work, it is important that patients are managed appropriately. Many surgeons understand the need to prevent valgus load to allow healing, but do not understand the importance of keeping the foot in neutral position to stop external rotation which, as the present study found, loads both the dMCL and sMCL.

Shapiro et al. [28] found that MCL deficiency caused a large increase of tension in the ACL when an external rotation was imposed: 10 Nm torque led to 55 N ACL tension in the intact knee, and 138 N with MCL deficiency. Therefore, unaddressed MCL deficiency leads to increased ACL graft tension, which might increase likelihood of failure. Persisting MCL laxity is associated with increased ACL graft revision [31]; at the lateral aspect, the addition of an extra-articular procedure reduces ACL graft failure rates [9, 30] and therefore, in the correct situation, an equivalent medial procedure could logically help. Furthermore, increased load on the ACL graft during the healing and remodelling phase may also result in an elongated non-functional ACL reconstruction which, whilst intact, may not allow the patient to return to the desired activities. The extra-articular procedures on the lateral side may, in fact, work to protect the ACL graft during this healing phase. Revision rate is a crude measure and many patients have persistent instability which is not picked up by the registry, because the reconstructed ACL has not ruptured.

The posterolateral ligament complex also resists external rotation. The posterolateral structures were not included in this study related to AMRI, because they usually relate to fundamentally different situations. A clinical examination may use the dial test to look for increased external rotation. With AMRI, the pathological axis of rotation is in the lateral compartment, leading to a coupled anterior prominence of the medial plateau that results from loss of the medial restraints. Conversely, injury of the posterolateral structures usually relates to posterolateral rotatory instability (PLRI), in which the axis of rotation is shifted into the medial compartment and the lateral plateau and fibular head move posteriorly [1]. This distinction is important during clinical examination: while the dial test may be positive in both situations, addressing the MCL rather than PL structures is appropriate for AMRI. Of course, it is possible to have both AM and PL structures injured, but the multi-ligament injured knee is beyond the scope of the present study.

The sMCL is the primary restraint of valgus loading across the arc of knee flexion. The percentage contribution of the sMCL increased from 0° to 30° flexion, due to the falling contribution of the PMC + POL, which slackens rapidly as the knee flexes [25, 35]. This reciprocal behaviour was also reported by Grood et al. [11]. The similarities between their results and the present study were despite differences in the testing method (a fixture in a materials testing machine versus a six degrees-of-freedom robot) and also the level of loading applied. Both the ACL and dMCL were secondary restraints to valgus, each resisting ~10% of the load across 0° to 60° knee flexion.

This is the first study to measure the primary and secondary restraints at the medial side of the knee using a modern robotic testing system, but the findings must be interpreted in the light of the limitations inherent in any study in vitro: the loads applied to the knees were similar to those imposed during clinical manual laxity tests: the dial, Lachman and valgus stress tests, rather than the loads expected in functional activities, which would have influenced the stability of the knee. That was necessary to avoid stretching-out the remaining intact ligaments during the repetitive cutting and loading sequence. Previous work [11] used valgus moments of 25 Nm without reporting stretching-out, while the present study limited the valgus loading to 8 Nm. The tests could only discern the passive contributions to restraint, while the muscles remained unloaded. The sequential cutting protocol used in the present and some previous studies [26, 27] depends on there being a lack of significant load-transfer interactions between each of the structures of interest. Given that the components of the MCL and PMC do not twist around each other during knee flexion, but remain mechanically in parallel, that may be discounted in this study. Finally, the robot was run in displacement control, so that the tibial displacements of the intact joint were repeated even after the ligaments had been cut—that led directly to the data about the reduced restraining forces. It could not show the corresponding increases in joint laxity that are seen during clinical examination, but many papers have already provided such data.

Conversely, the strengths of this study include the repeated-measures testing protocol, which minimises any inter-specimen variability effects, and the ability of the robot testing system to apply repeatable tibial displacements and accurately measure resulting knee forces/torques. The specimens had a mean age of 38 years, similar to the patients who usually sustain combined ACL/MCL injuries that lead to AMRI.

The clinical importance of this work is that it shows why isolated ACL reconstruction may not restore intact knee laxity in the setting of a combined ACL plus MCL injury that causes AMRI. This accounts for some cases of ACL reconstruction failure [28, 31]. The roles of the sMCL and dMCL in resisting valgus and external rotation found in this study suggest that treatment of chronic laxity following a combined lesion may require the addition of a medial procedure to an ACL reconstruction. Persisting AMRI may increase the risk of further injury following isolated ACL reconstruction. This study highlights the importance of recognising the extent of the medial injury with resultant treatment pathways designed to protect the ACL graft and optimise the healing of the individual medial structures. It has shown the role of the dMCL in restraining tibial external rotation when the knee is at/near extension, and the increasing role of the sMCL in resisting external rotation with knee flexion. In the clinic, dMCL and sMCL injury will cause increased external rotation in the dial test, but that must be identified carefully as being AMRI and not PLRI. It has also quantified the contributions of the ACL and components of the medial ligament complex when they act as secondary restraints, including the ACL resisting valgus, and the POL resisting internal rotation and valgus near knee extension. This work has provided data of direct usefulness in clinical work, by showing which of the medial-side structures are the most important to address when abnormal anteromedial knee joint laxity has been found.

## Conclusion

The dMCL and sMCL are the most important medial structures for resisting tibial external rotation, with the dMCL being the most important near full extension. Clinical symptoms caused by failure of conservative treatment of dMCL lesions [21], and the increase in ACL graft rupture with persistent AMRI, suggest further work to develop an MCL reconstruction method that includes the dMCL is needed. As expected, the sMCL is the most important restraint to valgus load. The ACL is the most important restraint to anterior draw and a secondary restraint of external rotation. The POL + PMC is a secondary restraint to valgus and both internal and external rotation near to full knee extension, but is slack in flexion.
